# Comments on Professor Peirce’s Paper, “Pyorrhœa Alveolaris”

**Published:** 1894-04

**Authors:** Edwin T. Darby

**Affiliations:** Philadelphia


### COMMENTS ON PROFESSOR PEIRCE'S PAPER, "PYORRHŒA ALVEOLARIS."[1]

BY EDWIN T. DARBY, M.D., D.D.S., PHILADELPHIA.

Your letter of February 5 came duly to hand. I am not yet prepared to commit myself to the theory which Dr. Peirce has advanced as to the cause of pyorrhœa alveolaris. I am free to admit that he has made suggestions which look wonderfully reasonable, and

---

[1] Read before the Eighth District Dental Society, Buffalo, N. Y., February 26, 1894.

I shall not be greatly surprised if before many months shall have passed away additional proof will be furnished which will confirm his theory. As yet he has not proved that uric acid is always present in the deposits to be found upon the roots of teeth in which pyorrhœa alveolaris has been known to exist, nor has he proved that uric acid is not present in the mouths of persons who have no disease of this character or in whom there is not known to exist a gouty diathesis.

Of late the disease known as gout is made to stand as godfather and sponsor for so many conditions that I sometimes fear we may overreach the mark and make it appear that nearly all our imperfections are the result of the uric-acid diathesis.

The medical profession of America is recognizing a peculiar condition of things in the American people which does not exist to the same extent in any other nationality, and which is doubtless the result of our mode of living, and which they have denominated "American gout." It is not the gout of England, which it has been thought was the result of port wine and beef, and which began in a man's big toe and made him wish he were dead, but it is the gout of malnutrition, and that is the result of a nervous exhaustion from overwork and *under*-feeding. I do not mean by this that food enough does not enter the system, for it does, but it is the inability of the system to properly appropriate that food, no matter what the character of it, that loads the system with uric acid. So far as I can learn, uric acid has never been found in the saliva; hence uric acid would not be found in *salivary* calculus, but it is nearly always present in the blood of gouty subjects, hence it might be found in the *serumal* deposits at the apex of the roots of teeth which have been lost by pyorrhœa alveolaris.

I am strongly of the opinion that Dr. Peirce is right, and I am also of the opinion that his description of the progress of the disease is the correct one. Pyorrhœa alveolaris is *not* a disease beginning at the gingivæ and the result of a local irritation, but a constitutional condition, and I trust that we shall soon know that it is accompanied with a gouty diathesis.

